# Knee Joint Loads and Surrounding Muscle Forces during Stair Ascent in Patients with Total Knee Replacement

**DOI:** 10.1371/journal.pone.0156282

**Published:** 2016-06-03

**Authors:** Robert Rasnick, Tyler Standifird, Jeffrey A. Reinbolt, Harold E. Cates, Songning Zhang

**Affiliations:** 1 Department of Kinesiology, Recreation, & Sport Studies, The University of Tennessee, Knoxville, Tennessee, United States of America; 2 Department of Exercise Science & Outdoor Recreation, Utah Valley University, Orem, Utah, United States of America; 3 Department of Mechanical, Aerospace, and Biomedical Engineering, The University of Tennessee, Knoxville, Tennessee, United States of America; 4 Tennessee Orthopedic Clinics, Knoxville, Tennessee, United States of America; Semmelweis University, HUNGARY

## Abstract

Total knee replacement (TKR) is commonly used to correct end-stage knee osteoarthritis. Unfortunately, difficulty with stair climbing often persists and prolongs the challenges of TKR patents. Complete understanding of loading at the knee is of great interest in order to aid patient populations, implant manufacturers, rehabilitation, and future healthcare research. Musculoskeletal modeling and simulation approximates joint loading and corresponding muscle forces during a movement. The purpose of this study was to determine if knee joint loadings following TKR are recovered to the level of healthy individuals, and determine the differences in muscle forces causing those loadings. Data from five healthy and five TKR patients were selected for musculoskeletal simulation. Variables of interest included knee joint reaction forces (JRF) and the corresponding muscle forces. A paired samples t-test was used to detect differences between groups for each variable of interest (p<0.05). No differences were observed for peak joint compressive forces between groups. Some muscle force compensatory strategies appear to be present in both the loading and push-off phases. Evidence from knee extension moment and muscle forces during the loading response phase indicates the presence of deficits in TKR in quadriceps muscle force production during stair ascent. This result combined with greater flexor muscle forces resulted in similar compressive JRF during loading response between groups.

## Introduction

Total knee replacement (TKR) is commonly used to correct end-stage knee osteoarthritis (OA) of the knee joint. The frequency with which TKRs are performed was expected to double in the US alone by 2015 and reach nearly 3.5 million by 2030 [1]. The primary purposes of a TKR are to alleviate pain, restore normal range of motion (ROM), and restore the ability to perform activities of daily living. Several studies have reported reductions in the pain after a TKR [2–4]. However other studies have reported disappointed patients due to post-surgery pain [5, 6] and difficulties with stair negotiation [6]. Difficulty with stair climbing prolongs a challenge to TKR patents, which a TKR is intended to correct. Stair climbing is a common activity of daily living, with older adults utilizing stairs as frequently as younger adults [7]. Additionally, stair climbing is utilized in all clinical recovery assessments after a TKR including the original knee society scoring system [8], the new knee society scoring system [9], and the oxford knee [10].

Experimental studies have been conducted to provide an understanding of how well a TKR actually restores a healthy gait in end-stage OA patients [11–15]. A recent review of stair ambulation after TKR reported that during stair ascent, peak knee extension moment appears to be reduced following TKR compared to healthy subjects [15], though this review only included studies utilizing an inverse dynamics approach to calculate joint moments. The information provided by net joint moments and net joint reaction forces via inverse dynamics does not provide a true bone on bone loading at the knee joint. Loading at the joint results from the contraction of muscles, not just the GRF propagated up through the body. Several studies have provided a good understanding of these effects by utilizing an instrumented TKR while climbing stairs [16–23]. Compressive loads at the knee during stair ascent were found to range between 2.5 and 3.06 times bodyweight (BW) [18–20]. Shear loads at the knee during stair ascent were also reported with rather large variability [17, 20, 22]. While instrumented implants do provide accurate joint loading information during stair ascent, they are limited in subject populations due to expenses and patient consent, and they do not provide the ability to examine muscle forces surrounding the knee joint.

Musculoskeletal modeling and simulation provides a means to approximate joint loading and corresponding muscle forces during a movement. Kim, et al. [21] made comparisons between musculoskeletal simulation and instrumented implant data, showing good agreement between contact forces of both components of the tibiofemoral joint during overground walking. Musculoskeletal simulation has been utilized in applications for overground walking in healthy and patient populations including knee OA and cerebral palsy [24–29]. Knee joint force has been reported in these studies to range from 2.8 BWs in healthy individuals to nearly 7 BWs in those with a severe crouch gait [24, 25, 27, 29]. However, only a limited number of studies have utilized musculoskeletal simulations to investigate knee joint loading in stair negotiation tasks [30–33]. Taylor et al. (2004) compared over-ground walking to a single step-up in total hip arthroplasty patients and found peak knee compressive force to range between 4.9 and 5.6 BWs during the step-up task. Complete understanding of loading to the knee for TKR populations is of great interest in order to aid rehabilitation, implant design improvements, patient education, and future healthcare research. Musculoskeletal simulation can provide an excellent means of determining true subject-specific knee loading and aid in improving TKR design and restoring TKR patients to normal loading and movement patterns.

To date, no studies have investigated the knee joint loading and muscle forces in TKR patients during stair ascent using musculoskeletal simulation. Simulation studies have only utilized a single step-up task in their analysis failing to represent the actual movement while climbing stairs. Knee joint loads and surrounding muscle forces during stair ascent for TKR patients are not clear. Therefore, the purpose of this study was to determine if the knee joint loadings following TKR are recovered to the level of healthy individuals, and determine the differences in muscle forces causing those loadings. Loading to a joint is greatly contributed by muscle forces and it is well documented that TKR knees show reductions in muscle forces compared with the knees of healthy individuals. Thus, it was hypothesized that knee joint compressive forces would be reduced following a TKR compared with those in healthy individuals, and that muscle forces causing knee extension would also be reduced. The posterior stabilized TKRs used in this study provides increased anterior/posterior stability. As such, it was further hypothesized that knee shear loading of the TKR participants would be reduced compared to healthy counterparts.

## Materials and Methods

### Participants

The University of Tennessee Institutional Review Board approved the study. All participants in the study signed a written informed consent prior to data collection. All participants were recruited for a larger study currently underway in our lab. TKR patients were recruited through a local orthopedic clinic and all surgeries were performed by the same surgeon. Five patients (63.6 ± 8.7 yrs, 1.74 ± 0.1 m, 87.0 ± 8.9 kg) received a posterior stabilized TKR and were 14.6 ± 3.4 months (eleven to nineteen months) post-surgery at the time of the data collection. Five healthy participants (57.8 ± 10.0 yrs, 1.78 ± 0.1 m, 89.0 ± 6.6 kg) had no knee pain in the past 6 months during daily activities and not been diagnosed of lower extremity joint OA. They were age, gender, height and body mass matched with TKR patients. Additionally, healthy participants were selected using the same exclusion criteria for TKR patients (Table 1).

**Table 1:** Inclusion and exclusion criteria for the TKR subjects.

Exclusion Criteria:		Inclusion Criteria:	
**-**	Any additional lower extremity joint replacement.	-	Men and women between the ages of 35 and 80.
**-**	Any lower extremity joint arthroscopic surgery or intra-articular injection within the past month.	-	Total knee replacement in one knee.
**-**	Systemic inflammatory arthritis (rheumatoid arthritis, psoriatic arthritis) (self-reported).	-	At least 6-months from TKR.
		-	No more than 5-years from TKR
**-**	BMI greater than 35.		
**-**	Inability to ascend/descend stairs without the use of a handrail.		
**-**	Neurologic disease (e.g. Parkinson’s Disease, stroke patients) (self-reported).		
**-**	Any major lower extremity injuries/surgeries.		
**-**	Inability to walk without a walking aid.		
**-**	Any visual conditions affecting gait or balance.		
**-**	Women who are pregnant or nursing.		
**-**	Any cardiovascular disease or primary risk factor which precludes participation in aerobic exercise as indicated by the Physical Activity Readiness Survey.		

### Experimental Procedures

**Fig 1:**
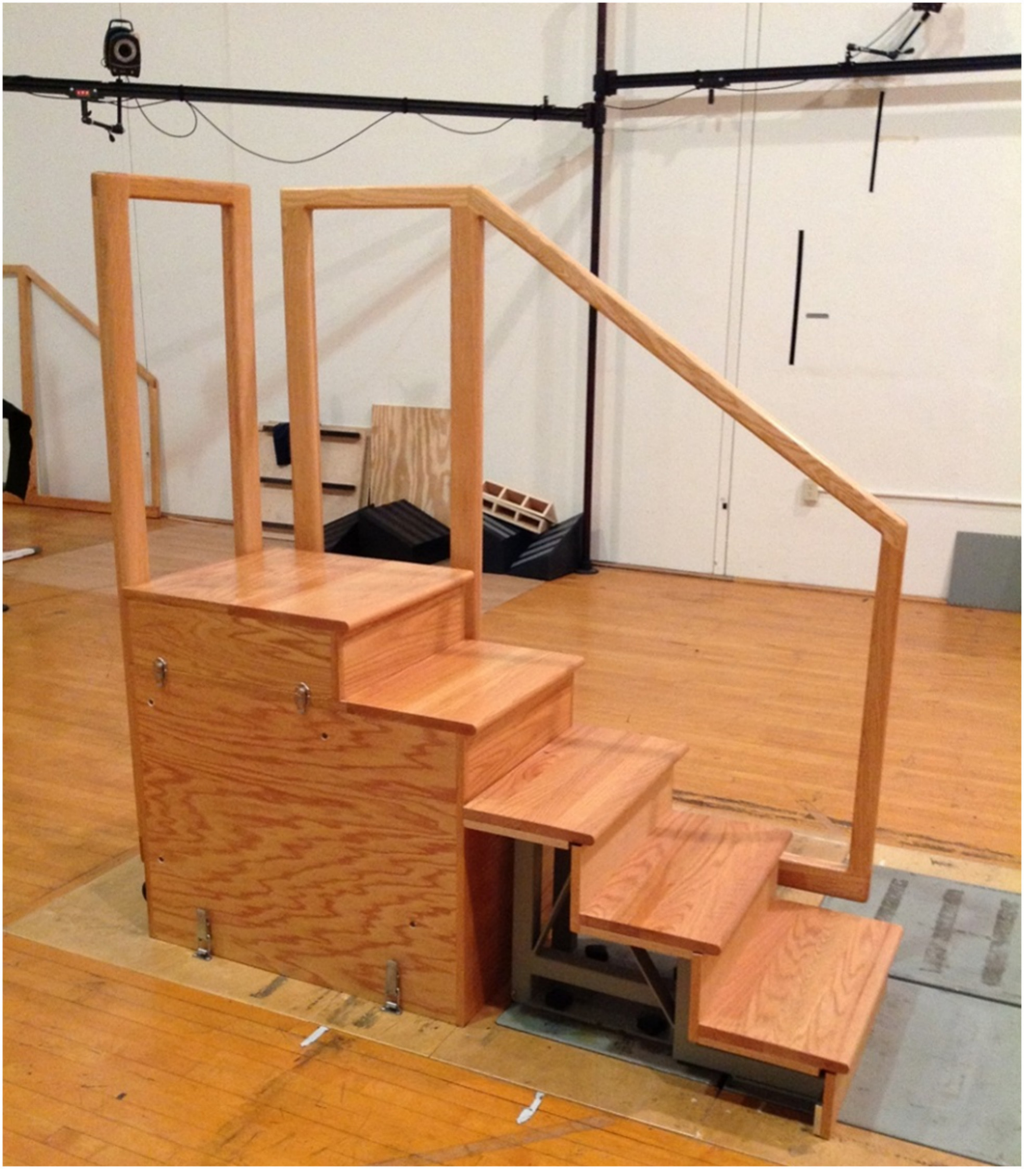
The 5-step (3 instrumented and 2 for turning around) staircase securely bolted to two force platforms for experimental data collection of ground reaction forces during stair negotiation.

Three dimensional kinematic data were collected experimentally using a nine-camera motion analysis system (240 Hz, VICON Motion Analysis Inc., Oxford, UK). Reflective anatomical markers were placed bi-laterally on the following anatomical landmarks: acromion processes, iliac crests, greater trochanters, medial and lateral femoral epicondyles, medial and lateral malleoli, 1^st^ and 5^th^ metatarsal heads, and toes (i.e. the most anterior aspect of the shoes). Reflective tracking markers connected to semi-rigid thermoplastic shells were secured to the trunk, pelvis, thighs, shanks, and on the posterior and lateral heel counter of a pair of standard lab shoes (Noveto, Adidas, USA). A three-step staircase (FP-Stairs, American Mechanical Technology Inc., Watertown, MA, USA) was securely bolted to two force platforms (1200 Hz, BP600600 and OR-6-7, American Mechanical Technology Inc., Watertown, MA, USA) in order to measure ground reaction forces (GRF) during stair negotiation (Fig 1). An additional two steps and a platform were also included [34, 35].

Before the reflective markers were positioned, participants walked for 3 minutes on a treadmill at a self-selected pace as a warm-up. All participants then performed functional assessments including: timed up and go (TUG) [36], knee range of motion (ROM) [37], and stair ascent and stair descent times [38]. Participants were asked to perform a minimum of three practice trials to determine their self-selected speeds. Participants then performed five successful trials of stair ascent at the self-selected speed (±5%) which was monitored by two photo cells and electronic timers (Lafayette Instrument Inc., IN, USA). Step two was the step of interest [39].

### Data Analyses

Visual 3D (C-Motion, Inc., Germantown, MD, USA), a biomechanical analysis software suite was used to filter both kinematic and ground reaction force data at 8 Hz [40], respectively, using a fourth-order Butterworth low-pass filter. An X-Y-Z (for rotation about mediolateral, anteroposterior and longitudinal axes) Cardan rotational sequence was used in joint angle calculations and the right hand rule was used for determining the conventions for joint kinematics and kinetics. All joint moments were computed as internal moments.

### Musculoskeletal Modeling and Simulations

The processed individual trials were exported to OpenSim (version 3.0.1, SimTK, Stanford, CA, USA) to perform musculoskeletal simulations. A generic 12-segment, 19-degree of freedom, and 92 muscle-actuated OpenSim musculoskeletal model (Gait 2392 Model), originally developed by Delp, et al. [41], was scaled to the height and weight of each individual participant to generate subject-specific models. In order to improve the accuracy of the simulations a residual reduction algorithm (RRA) was used to minimize virtual residual forces added to the model to account for dynamic inconsistency as results of experimental errors and modeling assumptions by making small adjustments to the joint accelerations and body mass parameters [42]. Kinematic changes from RRA were all kept below 5.5 cm of translation and 3.5 degrees of rotation; peak residual forces and moments were each kept below 14% of body weight and 1.6 Nm/kg, respectively [43]. Though slightly higher than what is recommended (43), these do not greatly influence the comparisons between our two participant groups and values for joint angles, moments and joint reaction forces were ensured to be within two standard deviations of previous literature, consistent with the suggested practices for the validation of musculoskeletal models [23, 43]. Individual muscle excitations and resulting muscle forces were calculated using computed muscle control (CMC) to drive simulations of the stair ascent movement collected experimentally [44, 45]. The CMC, as a part of static optimizations, has been shown to generate tibiofemoral contact forces that are higher than those measured in vivo, but have similar timings and magnitudes [46]. Additionally, the utilization of CMC has a strength over static optimization alone due to the ability to account for the residuals at the pelvis obtained in RRA by numerical integration, which allows for the residuals to be linked together temporally with the original movements, providing more accurate simulation. Joint reaction forces (JRF) were computed using the JRF Tool in OpenSim.

The dependent variables included: peak vertical GRF, peak knee extension moment, peak knee abduction moment, peak knee compressive force, peak knee anterior shear forces, peak knee extensor and flexor muscle forces, velocity, and the functional assessments. The stance of gait was divided into early stance (loading) and late stance (propulsion) phases, which was divided at about 50% of stance phase based upon the time when the anteroposterior GRF changed from a negative value to a positive value. Peak values of forces and moments were reported for the two phases separately. The average of each variable for the five stair ascent trials of each subject was used in the statistical analyses. In order to compare differences between TKR patients and healthy individuals, a paired samples t-test was used for each variable (21.0, IBM SPSS, Chicago, IL) with an alpha level set at 0.05 a priori.

## Results

**Fig 2:**
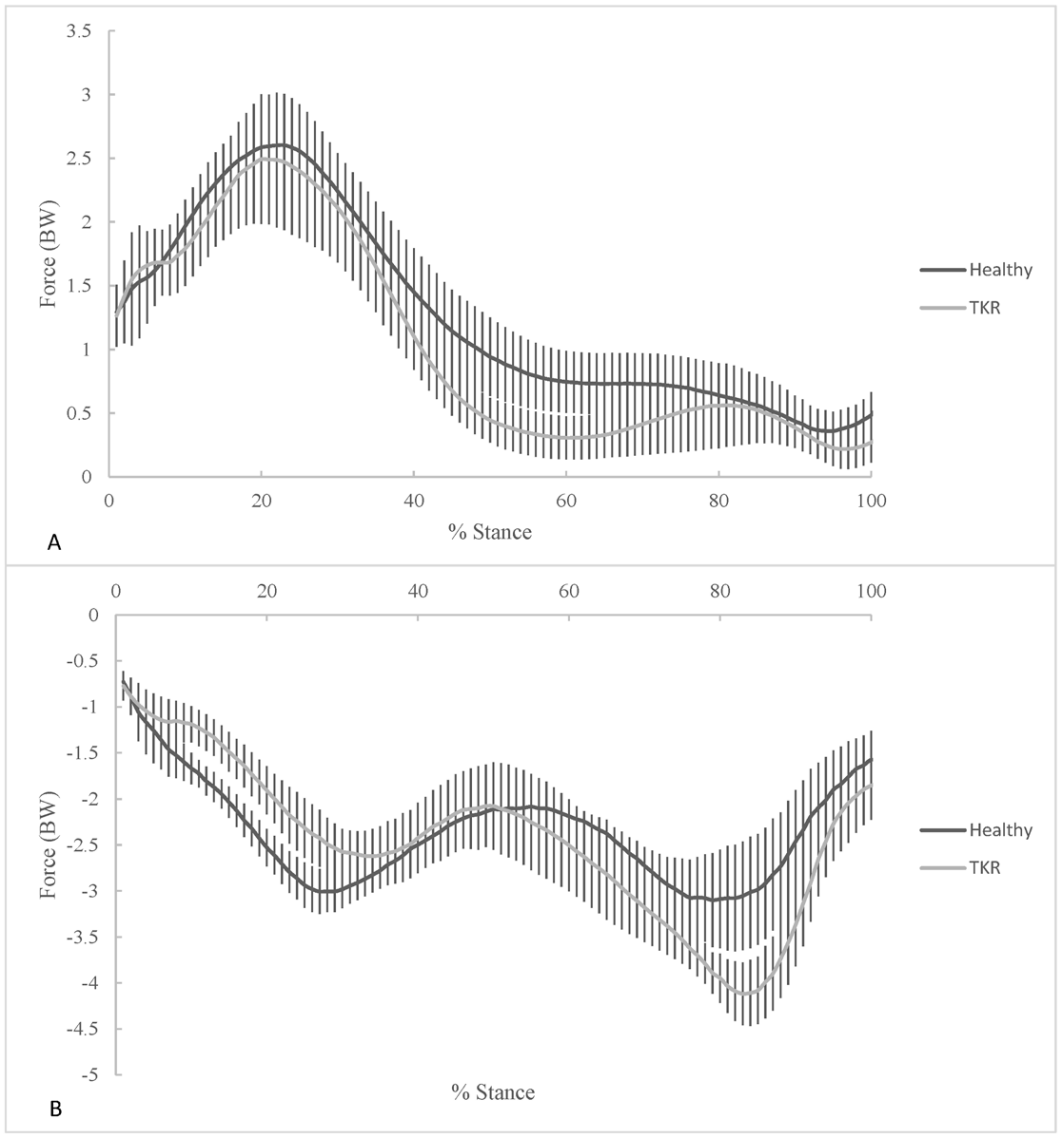
Comparison of mean knee joint reaction forces (JRF) during stair ascent for healthy controls and TKR patients. (A) shear and (B) compressive were computed using the joint reaction force (JRF) tool in OpenSim.

No significant differences in age (p = 0.358), height (p = 0.540), and mass (p = 0.688) existed between TKR patients and healthy subjects. No significant differences were found in stair ascent velocity (actual velocity obtained during movement trials), knee ROM, TUG, and stair ascent time between healthy controls and TKR patients (Table 2). There were no differences between groups for knee flexion angle at contact (p = 0.302) or knee extension ROM during the stance phase (p = 0.645) (Table 3). The peak knee extensor moment was similar in TKR patients and healthy controls (p = 0.174, Table 3). The knee JRF curves demonstrate an early stance peak for the shear component and a smaller early stance peak and a larger late stance peak for the compressive component (Fig 2). No differences were found between groups for compressive joint reaction forces.

**Table 2:** Stair ascent velocity and functional assessments of healthy controls and TKR patients (Mean ± SD).

	Healthy	TKR	P-value
**Velocity (m/s)**	1.6 ± 0.2	2.1 ± 0.6	0.154
**Knee ROM (deg.)**	121.4 ± 7.4	113.6 ± 7.3	0.133
**TUG (sec.)**	7.4 ± 1.2	7.4 ± 0.5	0.991
**Stair Ascent Time (sec.)**	6.2 ± 0.2	7.0 ± 0.7	0.055

**Table 3:** Peak GRF, knee angle, knee moments, and knee JRF of healthy controls and TKR patients during stair climbing (Mean ± SD).

Variable	Healthy	TKR	P-value
**Early stance Peak Vertical GRF BW)**	1.03 ± 0.05	0.98 ± 0.04	0.133
**Late Stance Peak Vertical GRF (BW)**	1.15 ± 0.16	1.09 ± 0.11	0.456
**Knee Flexion Angle at Stair Contact (Deg)**	67.5 ± 5.00	64.7 ± 2.81	0.302
**Sagittal Knee Range of Motion (Deg)**	57.5 ± 3.34	58.9 ± 5.70	0.645
**Peak Extension Moment (Nm/kg)**	1.07 ± 0.20	0.87 ± 0.23	0.174
**Early Stance Peak Abduction Moment (Nm/kg)**	0.26 ± 0.21	0.36 ± 0.25	0.518
**Late Stance Peak Abduction Moment (Nm/kg)**	0.19 ± 0.12	0.28 ± 0.13	0.266
**Peak Anterior Shear JRF (BW)**	2.82 ± 0.47	2.48 ± 0.50	0.299
**Early Stance Peak Compressive JRF (BW)**	-3.20 ± 0.34	-2.76 ± 0.36	0.089
**Late Stance Peak Compressive JRF (BW)**	-3.90 ± 0.54	-3.89± 0.65	0.988

**Fig 3:**
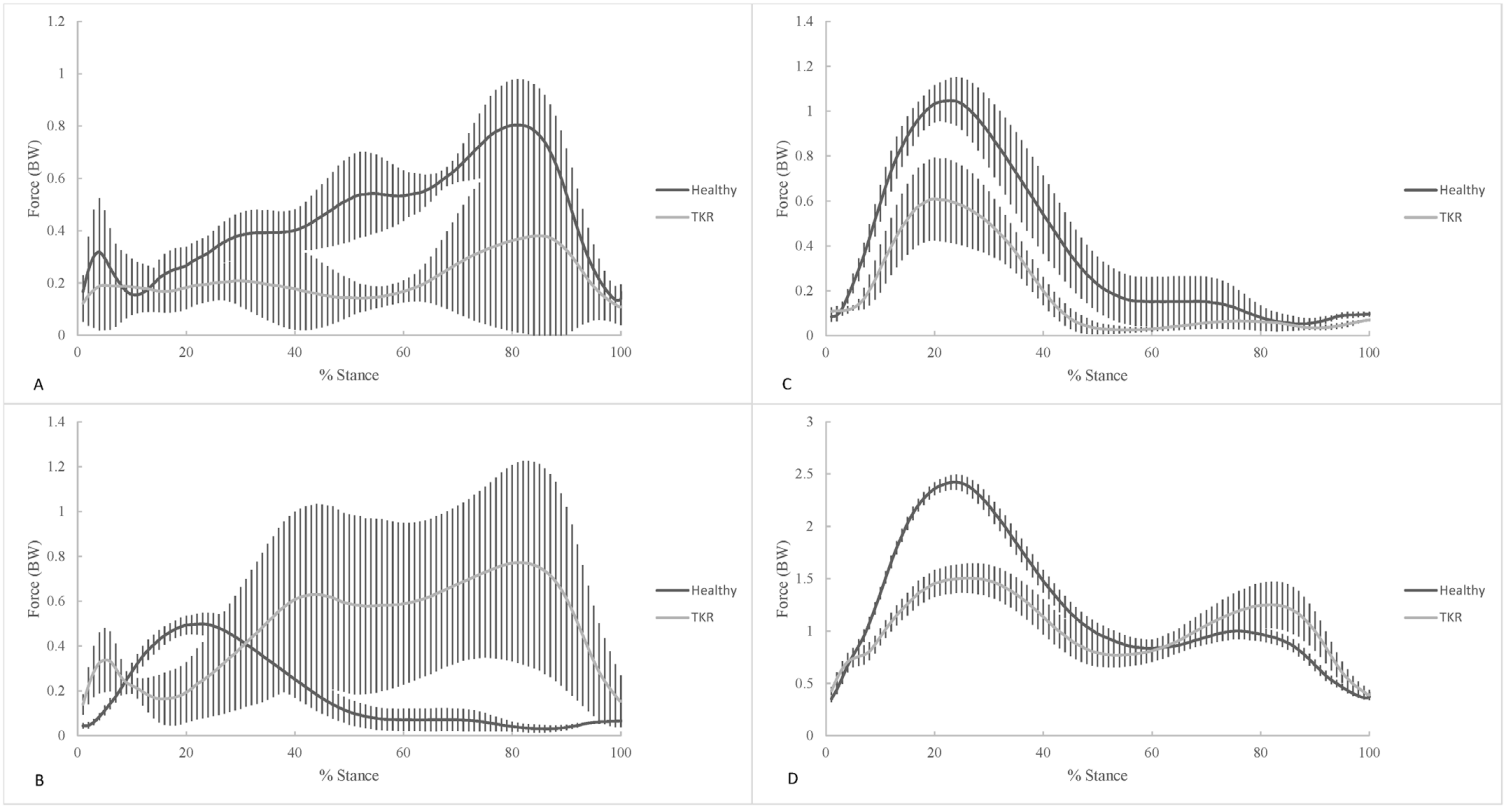
Comparison of mean knee extensor muscle forces during stair ascent for healthy controls and TKR patients. (A) rectus femoris, (B) vastus medialis, (C) vastus lateralis, and (D) sum of knee extensors were computed using the computed muscle control (CMC) tool in OpenSim.

The rectus femoris muscle force showed a smaller early stance peak and a larger late stance peak (Fig 3a) while the vastus medialis muscle had an early stance peak for TKR patients and a late stance peak for healthy controls (Fig 3b). The vastus lateralis muscle force had a large early stance peak (Fig 3c) and the sum of knee extensor muscle forces had a large early stance peak and smaller late stance peak (Fig 3d). The early stance peak muscle forces of the rectus femoris (p = 0.020), vastus lateralis (p = 0.003), and sum of knee extensor forces (p = 0.001) were reduced in TKR patients compared to healthy individuals (Table 4, Fig 3). The late stance peak muscle force of the vastus lateralis (p = 0.043) was reduced in TKR patients compared to their healthy counterparts (Table 5, Fig 3). However, the late stance peak vastus medialis force was greater in TKR patients compared to healthy controls (p = 0.010).

**Table 4:** Early stance peak knee extensor and flexor muscle forces for healthy controls and TKR patients during stair ascent (Mean ± SD).

Muscle Group	Muscle	Force (BW)		P-value
		Healthy Controls	TKR Patients	
**Knee Extensors**	**Rectus Femoris**	0.62 ± 0.16	0.31 ± 0.18	**0.020**
	**Vastus Medialis**	0.51 ± 0.06	0.77 ± 0.37	0.150
	**Vastus Intermedius**	0.58 ± 0.07	0.49 ± 0.13	0.178
	**Vastus Lateralis**	1.06 ± 0.12	0.63 ± 0.20	**0.003**
	**Sum**	2.45 ± 0.24	1.62 ± 0.24	**0.001**
**Knee Flexors**	**Semimembranosus**	0.50 ± 0.14	0.45 ±0.09	0.487
	**Semitendinosus**	0.08 ± 0.04	0.05 ±0.01	0.187
	**Bicep Femoris Long Head**	0.32 ± 0.05	0.30 ± 0.12	0.773
	**Bicep Femoris Short Head**	0.33 ± 0.15	0.53 ± 0.12	0.053
	**Sartorius**	0.03 ± 0.01	0.07 ± 0.02	**0.009**
	**Gracilis**	0.01 ± 0.007	0.03 ± 0.02	**0.041**
	**Medial Gastrocnemius**	0.73 ± 0.30	0.94 ± 0.27	0.296
	**Lateral Gastrocnemius**	0.22 ± 0.40	0.40 ± 0.14	0.060
	**Sum**	1.64 ± 0.47	1.83 ± 0.40	0.512

**Table 5:** Late stance peak knee extensor and flexor muscle forces for healthy controls and TKR patients during stair climbing (Mean ± SD).

Muscle Group	Muscle	Force (BW)			P-value
		Healthy Controls	TKR Patients		
**Knee Extensors**	**Rectus Femoris**	0.88 ± 0.19		0.41 ± 0.44	0.061
	**Vastus Medialis**	0.11 ± 0.05		0.86 ± 0.49	**0.010**
	**Vastus Intermedius**	0.13 ± 0.06		0.08 ± 0.03	0.115
	**Vastus Lateralis**	0.24 ± 0.13		0.09 ± 0.04	**0.043**
	**Sum**	1.19 ± 0.32		1.32 ± 0.29	0.507
**Knee Flexors**	**Semimembranosus**	0.42 ± 0.06		0.53 ± 0.14	0.156
	**Semitendinosus**	0.05 ± 0.02		0.05 ± 0.02	0.890
	**Bicep Femoris Long Head**	0.18 ± 0.09		0.15 ± 0.03	0.629
	**Bicep Femoris Short Head**	0.38 ± 0.06		0.47 ± 0.09	0.076
	**Sartorius**	0.04 ± 0.01		0.08 ± 0.02	**0.007**
	**Gracilis**	0.01 ± 0.004		0.01 ± 0.002	0.737
	**Medial Gastrocnemius**	1.02 ± 0.19		0.32 ± 0.42	**0.009**
	**Lateral Gastrocnemius**	0.31 ± 0.09		1.06 ± 0.47	**0.008**
	**Sum**	1.62 ± 0.23		1.89 ± 0.17	0.070

**Fig 4:**
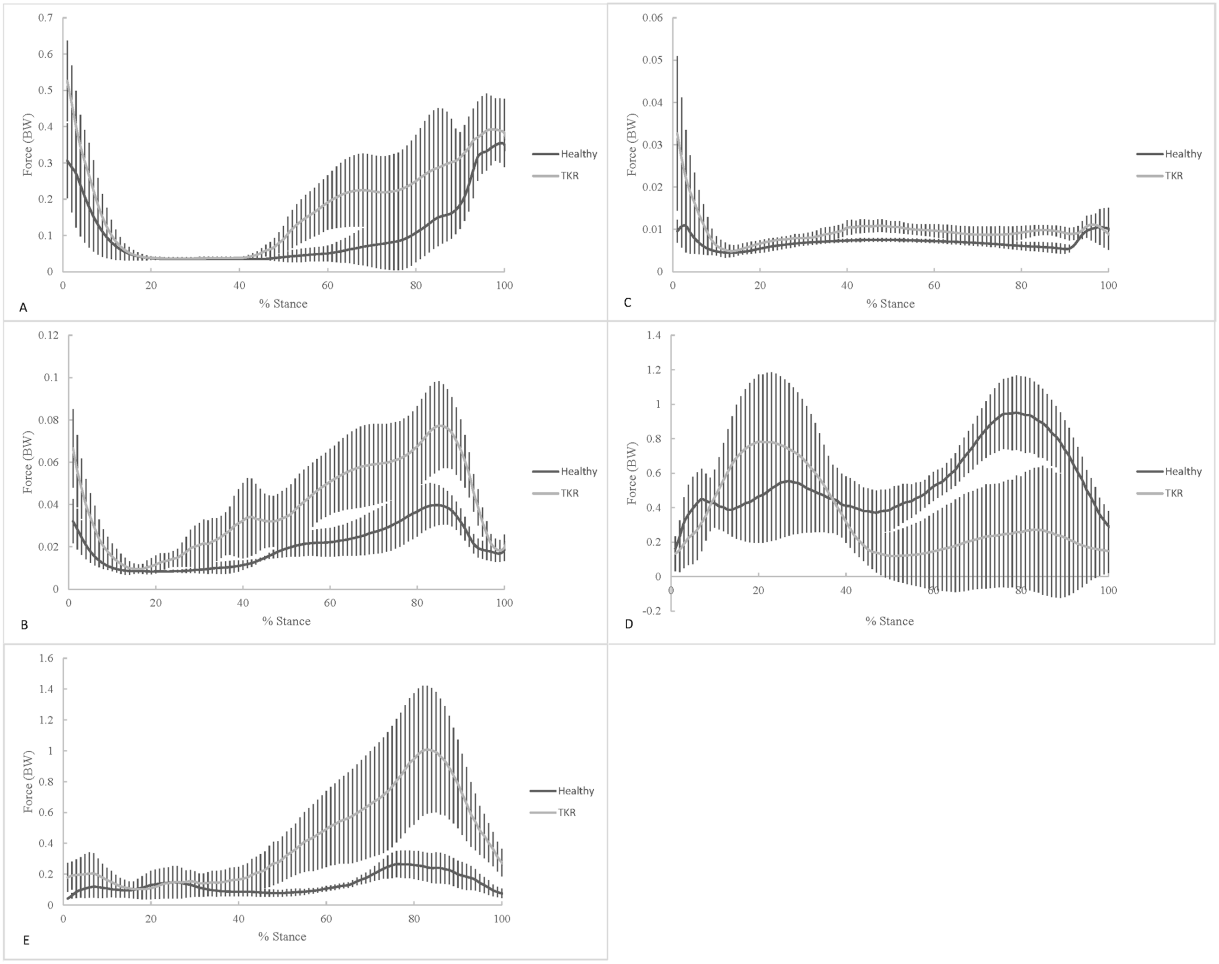
Comparison of mean knee flexor muscle forces during stair ascent for healthy controls and TKR patients. (A) short head of biceps femoris, (B) sartorius, (C) gracilis, (D) medial gastrocnemius, and (E) lateral gastrocnemius were computed using the computed muscle control (CMC) tool in OpenSim.

The biceps femoris muscle (Fig 4a) and sartorius muscle (Fig 4b) forces showed peaks at the beginning and end of stance phase. The gracilis muscle force had one large peak at the beginning of stance phase in TKR patients, while the force in control subjects was relatively constant (Fig 4c). The medial gastrocnemius muscle force showed a large first peak and a smaller second peak in TKR patients, while the force in healthy patients has a smaller early stance peak with a larger late stance peak (Fig 4d). Finally, the lateral gastrocnemius muscle force showed a late stance peak (Fig 4e). The early stance peak muscle force of the sartorius (p = 0.009) and gracilis (p = 0.041) were greater in TKR patients compared to healthy controls (Table 4, Fig 4). The late stance 2^nd^ peak muscle force of the sartorius (p = 0.007) and lateral gastrocnemius (p = 0.008) were found to be greater while the medial gastrocnemius (p = 0.009) was reduced in TKR patients compared to healthy controls (Table 5, Fig 4).

## Discussion

The primary purpose of this study was to determine the extent to which knee joint loading following TKR is recovered to the level of healthy individuals, and determine the differences in muscle forces causing those loadings. Our hypothesis was that knee joint compressive forces would be reduced in TKR knees as a result of reduced knee extensor muscle forces and that shear loading would also be reduced as a result of the posterior stabilized TKR knees. Our findings suggest that knee extensor muscle forces were reduced in TKR knees compared to healthy knees, but this did not lead to differences in compressive forces. Furthermore, the findings did not show a difference in shear loading between TKR and healthy knees.

The peak knee extensor moments in TKR patients compared to healthy individuals during stair climbing in this study were similar and contrary with findings reported in the literature [47–50]. This study reported that there were no differences between sagittal plane knee angles at contact or total range of motion, suggesting both groups had similar locations of the knee joint center throughout the stair climbing task. Pairing this with similar GRF values suggest that knee extensor moments would be similar between groups. Additionally, the results showed no differences in peak knee abduction moment, which are supported in the literature [47–49]. However, it becomes important to note that implant design has been shown to play a role in the existence of differences in peak knee abduction moment. Some studies have found differences between controls and TKR patients in peak knee abduction moment when using a mobile bearing design [48, 49]. All patients in this study received a posterior stabilized TKR, which has been shown to result in no differences in peak abduction moment in the literature [47–49].

The early stance peak compressive JRF was not different between groups, contrary to our hypothesis. Increased knee muscle forces are likely to be present in order for the compressive loading to be the same between groups as the loading response peak knee extensor moment was not different. In support of the hypothesis, combined knee extensor muscle forces during the loading response phase were reduced in TKR patients compared to healthy controls (Table 4). The peak rectus femoris and vastus lateralis forces were both reduced in TKR patients resulting in a reduced sum of knee extensor muscles. Interestingly, two accessory muscle forces, the sartorius and gracilis, also were greater in TKR patients. These findings complement the lack of differences in early stance peak compressive JRF. These results suggest that the differences between TKR patients and healthy controls during the loading response phase are primarily due to the differences in muscle forces. It might be assumed that differences in muscle force production would directly result in differences in the JRF, but clearly this is not the case. In addition, the TKR patients in this study had functional outcomes that were similar to healthy controls (Table 2). This finding suggests that while the reduced quadriceps forces may cause deficiencies in knee extensor moment production, this may not mean physical function is impaired.

Contrary to the hypothesis, the late stance peak compressive JRF were also found to be similar between groups. The magnitudes for compressive loading seen in this study were elevated slightly over those seen in the literature for stair ascent [17–20], but still within two standard deviations of previous instrumented studies. TKR patients were found to have an early stance peak compressive loading of 2.76 ± 0.34 BW and late stance peak of 3.89 ± 0.36 BW compared to instrumented implant literature which ranged from 2.5 to 3.06 BW [18–20]. Differences in velocity could explain the discrepancy between the findings reported here and those seen in the literature. The present study found no differences in the velocity of TKR patients compared to healthy individuals. However, velocity has been shown in the literature to be reduced in TKR patients compared to healthy controls [50]. Velocity has not previously been reported in the instrumented implant research [18–20] making it difficult to know if varying velocities are present in these studies. Instrumented implant studies are also limited in that they only have limited number of subjects.

During the push-off (second 50%) of stance, the results showed more variable differences in individual muscle forces than the loading response phase. While there were some increases and some reductions in muscle force for knee extensors and flexors neither the sum for flexors or extensors showed any group differences during the push-off phase. While no differences existed in peak values it appears a different strategy was utilized by TKR patients to produce similar levels of muscle forces compared with to healthy controls. It can be seen that the majority of muscle force during the push-off phase is from the rectus femoris in healthy individuals (Fig 3a). However, TKR patients utilized the vastus medialis more during push-off than healthy controls (Fig 3b). Similarly, TKR patients utilized the medial and lateral gastrocnemius differently than healthy individuals. On the other hand, healthy controls employed the medial gastrocnemius more during the second half of stance while TKR patients primarily used the lateral gastrocnemius more (Fig 4a and 4b). The underlying biomechanical factors causing these differences remain unclear and the influence they have on JRF. TKR patients may be utilizing the muscles differently as a compensatory strategy for the reduced knee extensor strength that remains after rehabilitation. It is possible that gait compensation strategies seen in knee OA patients to relieve pain linger after the TKR rehabilitation is completed. Knee extensor muscle strength has been shown to be reduced in OA patients [51]. Based on the findings of this study and others [47–50], the TKR or rehabilitation may have not restored the ability of TKR patients to produce normal levels of muscle force during stair climbing. Additional differences may exist at the ankle (not investigated here) resulting in the differences seen during the push-off phase of stance. It is quite possible that TKR patients utilized the plantarflexors more to climb stairs instead of the knee extensors. The greater medial gastrocnemius force seen during push-off seems to support this strategy. Future research should investigate the differences between healthy and TKR patients during stair ascent.

Peak shear JRF did not differ between healthy controls (2.82± 0.47 BW) and TKR patients (2.48 ± 0.50 BW) in the present study. However, shear loading in TKR patients was elevated above the findings seen in the literature for modeling of a single step-up task which range between 1.1 and 1.5 BW [33]. The findings of the present study suggest that TKR patients and healthy controls produce similar anterior shear loading and pattern during stair climbing (Table 3 and Fig 2). Differences between the present study and studies investigating a step up task may be due to differences in speed and innate differences between a step-up task and stair ascent.

Although the generic musculoskeletal model was scaled individually based dimensions of participants, it does not reflect actual details of musculoskeletal systems of the actual participants. The use of the same musculoskeletal model for both the healthy and TKR patients is a limitation which assumes that muscle geometry would be the same following a joint replace. It should be noted that following replacement the knee is different from a traditional knee, especially the wrapping point(s) of the quadriceps femoris around the patella, which might have influenced the magnitude of the moment arm and force of the quadriceps muscle and knee joint reaction forces. Future research on effects of TKRs on changes in quadriceps muscle moment arm, its force capacity and knee joint reaction forces is warranted. The validation of a musculoskeletal model is always an important aspect of modeling. The model of the current study was validated according to best practices outlines in previous research and contained a comparison to instrumented data. According to these practices our model is sufficient, but as always modeling does make assumptions. The same model was utilized for both groups and so differences to absolute values may be present, but they would be present in both groups and as such the relative differences in muscle and joint reaction forces would be consistent. Additionally, we only examined effects of muscle forces on joint reaction forces and not their effects on joint moments, though the overall knee joint moments are presented in both the sagittal and frontal planes in the current study. Furthermore, healthy older adults were assumed to have no radiographic knee osteoarthritis.

## Conclusions

Evidence from knee extension moment and muscle forces during the loading response phase indicates reduced muscle force during stair ascent in the knee extensors of TKR patients. This result combined with greater flexor muscle forces resulted in similar compressive JRF during loading response between groups. Some muscle compensatory strategies appear to be present in the push-off phase. Future research utilizing musculoskeletal modeling and simulation is necessary to investigate differences in muscle forces dependent on rehabilitation strategies and differences existing at the ankle. Also, different TKR designs always have a potential impact on joint loading and muscle contributions. Lastly, comparisons of pre- and post-surgery data would also provide more insights into understanding if knee joint loading and muscle force production is improved following joint replacement. Individual subject data can be found in S1 Table.

## Supporting Information

S1 Table Individual subject data. (XLSX)
